# Suppression of GATA-3 Nuclear Import and Phosphorylation: A Novel Mechanism of Corticosteroid Action in Allergic Disease

**DOI:** 10.1371/journal.pmed.1000076

**Published:** 2009-05-19

**Authors:** Kittipong Maneechotesuwan, Xin Yao, Kazuhiro Ito, Elen Jazrawi, Omar S. Usmani, Ian M. Adcock, Peter J. Barnes

**Affiliations:** Airway Disease Section, National Heart and Lung Institute, Imperial College, London, United Kingdom; University of Manchester, United Kingdom

## Abstract

**Background:**

GATA-3 plays a critical role in regulating the expression of the cytokines interleukin (IL)-4, IL-5, and IL-13 from T helper-2 (Th2) cells and therefore is a key mediator of allergic diseases. Corticosteroids are highly effective in suppressing allergic inflammation, but their effects on GATA-3 are unknown. We investigated the effect of the corticosteroid fluticasone propionate on GATA-3 regulation in human T-lymphocytes in vitro and in vivo.

**Methods and Findings:**

In a T lymphocyte cell line (HuT-78) and peripheral blood mononuclear cells stimulated by anti-CD3 and anti-CD28 in vitro we demonstrated that fluticasone inhibits nuclear translocation of GATA-3 and expression of Th2 cytokines via a mechanism independent of nuclear factor-κB and is due, in part, to competition between GATA-3 and the ligand-activated glucocorticoid receptor for nuclear transport through the nuclear importer importin-α. In addition, fluticasone induces the expression of mitogen-activated protein kinase (MAPK) phosphatase-1 (MKP-1), the endogenous inhibitor of p38 MAPK, which is necessary for GATA-3 nuclear translocation. These inhibitory effects of fluticasone are rapid, potent, and prolonged. We also demonstrated that inhaled fluticasone inhibits GATA-3 nuclear translocation in peripheral blood lymphocytes of patients with asthma in vivo.

**Conclusions:**

Corticosteroids have a potent inhibitory effect on GATA-3 via two interacting mechanisms that potently suppress Th2 cytokine expression. This novel mechanism of action of corticosteroids may account for the striking clinical efficacy of corticosteroids in the treatment of allergic diseases.

*Please see later in the article for Editors' Summary*

## Abstract 2

Peter Barnes and colleagues show that corticosteroids have a potent inhibitory effect on GATA-3 via two interacting mechanisms that suppress Th2 cytokine expression. This novel mechanism of corticosteroid action may help explain the efficacy of corticosteroids in allergic diseases.

## Editors' Summary

**Background:**

The immune system protects the human body from viruses, bacteria, parasites, and fungi. When one of these foreign invaders enters the body, immune system cells called T lymphocytes recognize specific molecules on the invader's surface and release chemical messengers (cytokines) that recruit and activate other types of immune cell, which then attack the invader. Sometimes, however, the immune system responds to a normally harmless material (for example, house-dust mites or grass pollen; scientists call these materials allergens) and triggers an allergic disease such as asthma or hay fever. Contact with an allergen activates a type of T lymphocyte called a T helper-2 (Th2) cell that subsequently makes (expresses) three cytokines called interleukin-4 (IL-4), IL-5, and IL-13. These cytokines ultimately cause inflammation (swelling) of the part of the body exposed to the allergen. Corticosteroids, which suppress the expression of cytokines by Th2 cells, are often used to treat inflammation in allergic diseases. Other treatments for these common conditions—about 50 million people in the US have an allergic disease—include minimizing exposure to allergens and diminishing the response of the immune system to allergens by using various immunotherapies.

**Why Was This Study Done?:**

Scientists know that corticosteroids reduce allergic inflammation by binding to proteins in immune system cells called glucocorticoid receptors. After binding to a corticosteroid, these receptors move into the nucleus of the cell (the part of the cell that contains its genes), where they suppress the expression of certain proinflammatory genes. However, it is still not known how corticosteroids inhibit the expression of Th2 cytokines. A key regulator of the expression of these cytokines and of allergic inflammation is a transcription factor called GATA-3. Transcription factors are proteins that control the expression of other proteins by binding to specific sequences in the genes that encode them. In this study, the researchers try to discover more about how corticosteroids reduce allergic inflammation by investigating the effects of the corticosteroid fluticasone on the regulation of GATA-3 activity in T lymphocytes.

**What Did the Researchers Do and Find?:**

Transcription factors have to move into the nucleus of cells (so-called nuclear translocation) to control the expression of their target genes, so the researchers first asked whether fluticasone affects the cellular localization of GATA-3. Fluticasone treatment of activated T lymphocytes growing in dishes, they report, inhibited the nuclear translocation of GATA-3 and reduced Th2 cytokine expression. Other experiments showed that the inhibition of GATA-3 nuclear translocation was partly caused by competition between the glucocorticoid receptor bound to fluticasone and GATA-3 for binding to importin-α, a protein that is required for nuclear import. However, fluticasone also prevented the nuclear translocation of GATA-3 in a second way. Before GATA-3 can bind to importin-α, phosphate groups have to be added to specific sites in GATA-3. This “phosphorylation” requires an enzyme called p38 MAP kinase, and the researchers found that fluticasone treatment of activated T lymphocytes induced the expression of MAP kinase phophatase-1, a p38 MAP kinase inhibitor. Finally, when the researchers treated seven patients with mild asthma with inhaled fluticasone, they found that fluticasone also inhibited GATA-3 nuclear translocation in the lymphocytes circulating in the patients' blood.

**What Do These Findings Mean?:**

These findings, obtained both in the laboratory and in patients, suggest that corticosteroids inhibit the expression of Th2 cytokines and thus reduce allergic inflammation through two interacting mechanisms. They suggest that corticosteroids prevent the nuclear translocation of GATA-3, a key regulator of Th2 cytokine expression, by competing with GATA-3 for binding to importin-α and by preventing the phosphorylation of GATA-3, a modification that allows GATA-3 to bind to importin-α. This dual mechanism of corticosteroid action may help to explain why these drugs are so effective in the treatment of allergic diseases, although further experiments are needed to show that the lymphocytes resident at sites of allergic inflammation respond to corticosteroids in the same way as lymphocytes in the blood. Finally, these findings suggest that the interaction between phosphorylated GATA-3 and importin-α might be a potential target for new treatments for allergic diseases.

**Additional Information:**

Please access these Web sites via the online version of this summary at http://dx.doi.org/10.1371/journal.pmed.1000076.

- The US National Institute of Allergy and Infectious Diseases provides information on allergic diseases and a simple description of the immune system
- The UK National Health Service Choices service provides information about allergies
- Links to other information about allergies are available from MedlinePlus (in English and Spanish)

## Introduction

Inflammation in allergic diseases such as asthma, rhinitis, and atopic dermatitis is mediated via expression of the cytokines interleukin (IL)-4, IL-5, and IL-13 from T helper-2 (Th2) cells. IL-4 and IL-13 regulate the expression of IgE from B lymphocytes, whereas IL-5 plays a key role in eosinophilic inflammation [1]. Th2 cytokines are regulated by the zinc finger transcription factor GATA-3, which is predominantly expressed in Th2 cells [2],[3]. GATA-3 determines Th2 cell differentiation and selectively activates the promoters of IL-4, IL-5, and IL-13 through chromatin remodelling [4]–[7]. The key role of GATA-3 in allergic airway inflammation has been demonstrated in mice by the reduced release of Th2 cytokines in animals treated with dominant-negative mutants of GATA-3 and by local application of antisense oligonucleotides to GATA-3 [8],[9]. Furthermore, conditional knock-out of the *Gata3* gene in mice reduces expression of Th2 cytokines in vitro and in vivo [10], and similar results have been reported in isolated murine CD4^+^ lymphocytes [11]. Finally, knockdown of *GATA-3* expression using siRNA in human T cells results in loss of anti-CD3/CD28-mediated Th2 cytokine expression [12].

In order for GATA-3 to regulate gene expression, it must translocate from the cytoplasm into the nucleus to access its target genes. Enhanced nuclear expression of *GATA-3* following T cell receptor activation was first demonstrated in murine T cells [13] and was recently confirmed in human T cells following T cell receptor and co-receptor stimulation [12]. GATA-3 contains a classical nuclear import signal [14] and is transported into the nucleus by the nuclear import protein importin-α (also known as karyopherin-α) [12]. Deletion of a region encompassing the GATA-3 nuclear localisation sequence (NLS) region in murine and human cells prevents its nuclear localisation [12],[14]. The affinity of the importin-α–NLS interaction is regulated by phosphorylation [15], and we have shown that p38 mitogen-activated protein kinase (MAPK) plays a critical role in phosphorylating GATA-3 to enhance its interaction with importin-α and subsequent transport into the nucleus [12].

Corticosteroids are highly effective in the treatment of allergic inflammation, with marked suppression of Th2 cytokines in airways of patients with asthma [16]. Corticosteroids mediate their anti-inflammatory effects through binding to glucocorticoid receptors (GRs), which then translocate to the nucleus where they interact with glucocorticoid response elements (GREs) in the promoter regions of steroid-sensitive genes. Alternatively, activated GR interacts with coactivator molecules to suppress the expression of inflammatory genes by inhibiting the action of proinflammatory transcription factors such as nuclear factor-κB (NF-κB) through the recruitment of co-repressor molecules such as histone deacetylase-2 [17],[18].

Nuclear localisation and retention of GR is mediated through the nuclear localisation sequences NL1 and NL2 [19], by nuclear retention signals [20], and by control of nuclear export via a chromosomal region maintenance 1 (CRM-1) dependent pathway [21]. NL1, which is similar to the SV40 NLS, binds to importin-α [22]. NL1 is activated both by glucocorticoid agonists such as dexamethasone and fluticasone propionate (FP) and by glucocorticoid antagonists such as mifepristone (RU486) [23]. NL1 can be mutated, and the resulting GR still translocates to the nucleus in response to ligands, but via interaction with importin 7, an event that requires an as-yet unknown component [23]. NL2 is poorly defined, residing in the ligand-binding domain, and much less is known about its mechanism of GR import [24]. A variety of other factors are also important for the regulation of GR activation and nuclear import including chaperones such as Hsp90 and other immunophilins [24]–[26] and FK506-binding proteins that may be linked to dynein and/or peptidylprolyl isomerase [27],[28].

However, the molecular basis for the inhibition of Th2 cytokines by corticosteroids is not well understood, because the genes encoding IL-4, IL-5, and IL-13 do not have any recognisable GRE sequence [29] and are only partly regulated by NF-κB in human cells [30]–[32]. Using overexpression and CAT-reporter genes, Lavender and colleagues [33] have shown that GR reduced GATA-3-mediated IL-5 and -13 promoter activity in human CD4+ T cells. The authors postulated that local recruitment of GR may alter the ability of GATA-3 either to bind to its target site, to cause transcriptional up-regulation, or to maintain an environment that is permissive for transcription.

We therefore investigated the effects of a synthetic corticosteroid, FP, on GATA-3 phosphorylation and nuclear translocation in a T lymphocyte cell line (HuT-78) and in peripheral blood mononuclear cells activated by anti-CD3 and anti-CD28 antibodies in vitro. We also studied the effects of inhaled fluticasone therapy on GATA-3 subcellular localization in peripheral blood mononuclear cells (PBMCs) from patients with asthma.

## Materials and Methods

### Participants and Study Design

We studied patients with mild asthma who were not treated with inhaled corticosteroids who had been included in a previously reported double-blind, placebo-controlled, crossover study with FP [34]. Seven patients with mild asthma entered the study and were randomized to receive a single inhalation of FP (100 and 500 µg) or a matched placebo control via a spacer chamber, and the other treatment was given after a wash-out period of at least 6 d. Blood was taken for preparation of PBMCs at 1 and 2 h after drug administration. All patients gave informed consent and the study was approved by the Ethics Committee of the Royal Brompton and Harefield Hospitals NHS Trust. The clinical study was conducted before the requirement for Clinical Trial Registration.

### Antibodies and Reagents

The monoclonal antibodies against human CD3, CD28, GR, and importin-α were purchased from BD Biosciences (Oxford, United Kingdom). Rabbit antibodies against human GATA-3 (H-48) and GR (E-20, sc-1003) were obtained from Santa Cruz Biotechnology (Santa Cruz, California, United States), polyclonal rabbit antibodies against phospho-p38 MAP kinase and phospho-ATF-2 from Cell Signaling Technology (New England Biolabs, Hertford, UK), monoclonal antibody against phosphoserine (clone 4H4) from Affiniti Research Products (Exeter, UK), and antibodies against rabbit IgG-conjugated TRITC from Dako Cytomation (Cambridge, UK). The anti-GR antibody (Clone 41) from BD Transduction Laboratories (Oxford, UK) was used for Western blot analysis. All other reagents were purchased from Sigma (Poole, UK).

### Cell Culture and PBMC Isolation

A human T cell line (HuT-78) was purchased from ECACC European Collection of Cell Culture (Wiltshire, UK) were cultured as previously described [12]. PBMCs were isolated by density centrifugation over Ficoll-Hypaque (density, 1.077 g/ml; Amersham Biosciences, Amersham, UK) as previously described [34]. Cells were stimulated with anti-CD3/CD28 (1 µg/ml each) for 1 h at 37°C to stimulate Th2 cytokine release in the presence or absence of FP (10^−12^ to 10^−8^M). Cytospins were prepared and GATA-3 localization determined by confocal microscopy as previously described [12].

### Reverse Transcription PCR

Total RNA was extracted using lysis buffer (RNeasy kit; Qiagen, Crawley, UK). During RNA purification, genomic DNA was digested with RNase-free DNase (Amersham Biosciences). Next, 0.5 µg of total RNA was reversed transcribed using the avian myeloblastosis virus RT (Promega, Southampton, UK). For relative quantification, RT-PCR was carried out using cDNA probes. Primers for IL-4 were from Sigma-Genosys (Cambridge, UK). Sequences of GADPH used are as follows: forward 5′-CCACCCATGGCAAATTCCATGGC, reverse 3′-TCTAGACGGCAGGTCAGGTCCAC.

### Cell Fractionation, Immunoprecipitation, and Western Blot Analysis

Nuclear and cytoplasmic fractions were prepared as previously described [35]. Whole cell lysates were prepared in NP-40 lysis buffer (0.5% Nonidet P-40, 20 mM Tris-HCl [pH 7.5], 150 mM NaCl) in the presence of complete protease cocktail inhibitor. Lysates were centrifuged at 4°C for 10 min at 12,000 rpm in an Eppendorf microcentrifuge to remove cellular debris. Samples were then immunoprecipitated with either 10 µl of antibody against GATA-3 or importin-α using A/G agarose slurry in the presence of protease inhibitor using the Catch and Release methodology (Upstate Biotechnology, Lake Placid, New York, USA). Western blot analysis was performed using anti-GATA-3, anti-importin-α, anti-GR, anti-p-p38 MAP kinase, anti-p-ATF-2, and anti-p-serine. Immunoreactive proteins were detected using an enhanced chemiluminescence ECL kit (Amersham Biosciences).

### Chromatin Immunoprecipitation

Chromatin immunoprecipitation (IP) was performed as previously described [12] in HuT-78 T-cells with 2 µg of anti-GATA-3 (Santa Cruz Biotechnology), or isotypic immunoglobulin G as a non-specific control (Santa Cruz Biotechnology) overnight at 4°C. Promoter sequences were detected with PCR primers for the IL-5 promoter (−445 to +4): forward 5′-TTAATCTAGCCACAGTCATAG-3′ and reverse: 5′-TCATGGCTCTGAAACGTTCTG-3′. PCR was performed using a Hybaid Omnigene thermal cycler (Hybaid, Ashford, UK) with cycling parameters of 72°C for 10 min, 35 cycles at 94°C for 45 s, 52°C for 45 s, 72°C for 45 s.

### GR-GATA-3 In Vitro Competition Assay for Importin-α (Far-Western ELISA)

Immunoprecipitated importin-α (anti-importin-α, Santa Cruz Biotechnology) from HuT-78 cells was separated by SDS-PAGE and purified from the excised gel by electroelution [36]. Similarly, GATA-3 was isolated from nonstimulated HuT-78 cells or cells stimulated for 30 min with anti-CD3/CD28 and GR was isolated from FP (10^−8^ M, 30 min) stimulated HuT-78 cells. These proteins were subsequently refolded in glycine solution. 100 µl of importin-α solution (100 ng/ml in TBS) was added to 96-well plates coated with goat anti-importin-α antibody. After 1 h incubation, GATA-3 (100 ng/ml in TBS) from stimulated or unstimulated cells were added to the importin-α-coated wells. GR (10 or 100 ng/ml in TBS) from stimulated or unstimulated cells were added to some wells. After a further 1 h incubation, the plate was washed and incubated with primary antibodies (a mixture of rabbit anti-GATA-3 and mouse anti-GR, Santa Cruz Biotechnology) for 1.5 h, and then incubated with secondary antibodies. FITC swine anti-rabbit IgG (Dako, Cambridge, UK) was used for the detection of GATA-3, and rhodamine donkey anti-mouse IgG (Novus, Littleton, Colorado, USA) was used for the detection of GR. FITC and rhodamine levels were measured with a fluorescent micro-plate reader (Bioline, London, UK).

### NF-κB Activation

NF-κB binding activity in nuclear extracts was determined using an ELISA-based kit (Trans-AM p65, Active Motif, Rixensart, Belgium). In brief, 5 µg of nuclear extracts were incubated with a plate coated with an NF-κB consensus oligonucleotide. Plates were washed before addition of an anti-p65 antibody. Antibody binding was detected with a secondary HRP-conjugated antibody and developed with TMB substrate. The intensity of the reaction was measured at 450 nm.

### Immunofluorescence Staining

Immunofluorescence staining was performed as previously described [34]. All staining was performed at room temperature and under humidification. Cells were collected and cytospins prepared in a cytocentrifuge (Shandon II, Shandon, Runcorn, UK). Cells were permeabilized, blocked, and then incubated with GR (1∶50 E-20 Santa Cruz Biotechnology) or anti-GATA-3 (H-48; Santa Cruz Biotechnology) antibody for 1 h at room temperature. After three washes in phosphate-buffered saline (PBS), cells were incubated with tetrarhodamine isothiocyanate–conjugated goat anti-rabbit antibody (Dako). Cytospins were counterstained with 4′,6-diamidino-2-phenylindole dihydrochloride (DAPI), a fluorescent blue nuclear indole chromatin stain, and mounted in PBS:glycerol (50∶50). The immunopositive signal was characterised using laser scanning confocal microscopy on a Leica TCS NT/SP interactive laser cytometer equipped with confocal optics (Leica Microsystems, Wetzlar, Germany). To determine the specificity of the antibodies, rabbit serum immunoglobulin (Dako) and secondary antibodies without the primary were used as controls. Positively stained nuclei and total cells were counted (500) on each slide with the observer blinded to the treatment.

### GATA-3-GFP Construct

The GATA-3 clone (BC003070) complete cDNA was obtained from Invitrogen Life Technologies as a 5′- EcoRI/3′-XhoI insert of GATA-3 in the pOTB7 vector. GATA-3 was excised from pOTB7 using XhoI digestion and pEGFP-C2 (Clontech, Saint-Germain-en-Laye, France) was digested with BamHI. DNA was recovered by phenol extraction and ethanol precipitation, and both the GATA-3 fragment and the pEGFP-C2 vector blunt-ended by incubation with Klenow (Bioline Bio-27029) for 30 min at 37°C. Klenow was inactivated by incubation for 10 min at 75°C. DNA was recovered by phenol extraction and ethanol precipitation, and both the blunt-ended GATA-3 fragment and the GFP vector were subsequently digested with EcoRI before the 5′-EcoRI/3′–blunt end GATA-3 fragment was inserted into the 5′-EcoRI/3′–blunt ended GFP vector. Positive clones were confirmed by digestion and size analysis by 1% agarose gel electrophoresis and by sequencing.

### Transfection

HuT-78 cells were transfected with either EP8 or GFP vector only DNA using solution R, programme V-001 at a ratio of 3×10^6^ cells/4 µg DNA for 7–8 h in complete medium (10% bovine serum in RPMI1640+15 L-glutamine) according to the general Amaxa protocol for nucleofection. The medium was subsequently changed to 1% RPMI for 24 h before transfected cells were added to anti-CD3/CD28 treated wells and live cell videomicroscopy performed.

### Time-Lapse Microscopy

HuT-78 cells expressing GATA-3-GFP were maintained at 37°C in growth medium in a closed FCS2 perfusion chamber (Bioptechs, Butler, Pennsylvania, USA) combined with an objective heater (Bioptechs) on the stage a Zeiss Axiovert 200 microscope (Thornwood, New York, USA). Observations were made by 40×1.0 NA oil-immersion objective lens, and fluorescence and phase contrast images were gathered using a Hamamatsu ORCA-ER charged coupled device camera (Bridgewater, New Jersey, USA) driven by Openlab software (Improvision, Coventry, UK). Photographs were taken at 0, 30, 60, 120, and 240 min.

### Densitometric Analysis

Densitometry of ECL immunoblots was performed using Gelworks ID intermediate software (Ultraviolet Products, Cambridgeshire, UK). Briefly, immunoblots were scanned and gates were drawn tightly around each band. Background values from each lane were subtracted to normalize each measurement. The bands were quantified using the Gelworks software. All Western blots were exposed to film for varying lengths of time, and only films generating subsaturating levels of intensity were selected for densitometric evaluation.

### Statistical Analysis

Data from three or more independent experiments are presented as the mean±standard error of the mean (SEM), except where stated and were compared using GraphPad Prism 4 (GraphPad Software, http://www.graphpad.com). Results were analysed using one-way ANOVA with Newman-Keuls post test except for the data from the in vivo inhaled FP study, which was analysed by Friedman's test with subsequent Wilcoxson matched pair signed rank sum test. Data from this analysis are presented as a box-and-whiskers plot. Friedman's test was used as three matched measures were obtained using placebo, 100 µg, and 500 µg of inhaled FP, which had variable baseline levels. We did not assume a Gaussian distribution of the data due to the limited numbers of participants analysed (seven).The null hypothesis was rejected at *p*<0.05.

## Results

### The Effect of Corticosteroids on GATA-3 Nuclear Translocation and IL-4 mRNA

**Figure 1:**
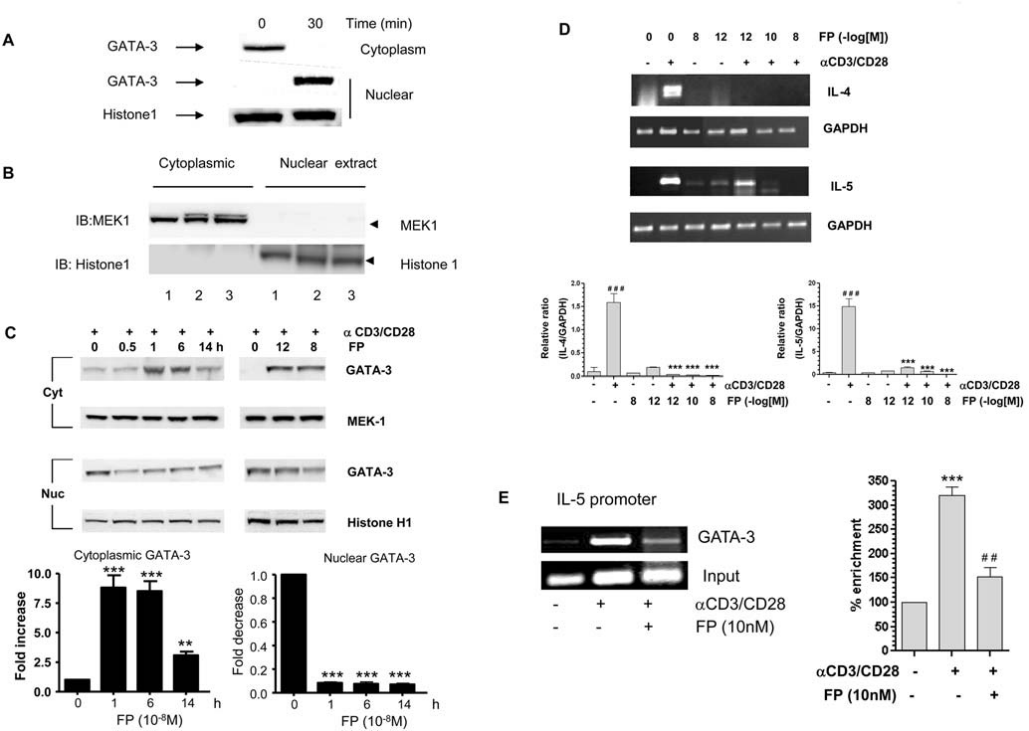
Fluticasone propionate down-regulates Th2 cytokine gene expression and inhibits GATA-3 nuclear import. (A) Anti-CD3/CD28 treatment of HuT-78 cells results in translocation of GATA-3 from the cytoplasm to the nucleus within 30 min. (B) Histone H1 and MEK-1 were used to confirm distinct separation of cytoplasmic and nuclear extracts in three separate experiments. (C) Western blot analysis of FP-treated HuT-78 cells demonstrated impaired nuclear localization of GATA-3 induced by anti-CD3/CD28 co-stimulation in a time- (at 10^−8^ M FP) and concentration- (at 60 min after stimulation) dependent manner. Cells were pretreated with FP for 30 min prior to stimulation. MEK1 and histone H1 were used to demonstrate equal cytoplasmic and nuclear loading respectively. Results are presented graphically below as mean±SEM of at least three independent experiments. *** *p*<0.001 compared to *t* = 0. (D) RT-PCR showing that FP inhibits IL-4 and IL-5 mRNA expression in CD3/CD28-costimulated cells. GAPDH was used as a loading control. Lower panels show graphical analysis of results presented as mean±SEM of at least three independent experiments. ^###^ *p*<0.001 compared to control, ***p<0.001 compared to anti-CD3/CD28–stimulated. (E) FP (10 nM) reduces the ability of anti-CD3/CD28-stimulated GATA-3 to associate with the native IL-5 promoter 60 min after stimulation. Data are also shown graphically as mean±SEM of three independent experiments. All data were analysed by ANOVA followed by Newman-Keuls post-test.

Corticosteroids are effective in inhibiting GATA-3-regulated IL-4 gene expression in vitro and in vivo [32]. We therefore investigated whether corticosteroids affect anti-CD3/CD28–stimulated nuclear import of GATA-3. Stimulation of cells with anti-CD3/CD28 resulted in a rapid cytoplasmic/nuclear GATA-3 translocation (Figure 1A), confirming our previous results [12]. We also confirmed a clear separation of nuclear and cytosolic fractions as indicated by histone H1 and MEK-1 markers (Figure 1B). The potent topical corticosteroid FP caused sustained loss of nuclear GATA-3 expression and cytoplasmic retention of GATA-3 at concentrations ranging from 10^−12^ to 10^−8^ M, which cover the therapeutic range [37]. This effect was concentration- and time-dependent, with a peak effect of 11.6-fold at 30 min at a concentration of 10^−8^ M (Figure 1C) and was associated with marked reductions in anti-CD3/CD28–stimulated IL-4 and IL-5 mRNA expression (Figure 1D) and a loss of GATA-3 binding to the native IL-5 promoter (Figure 1E).

### Ligand-Activated GR Competes with GATA-3 for Importin-α

**Figure 2:**
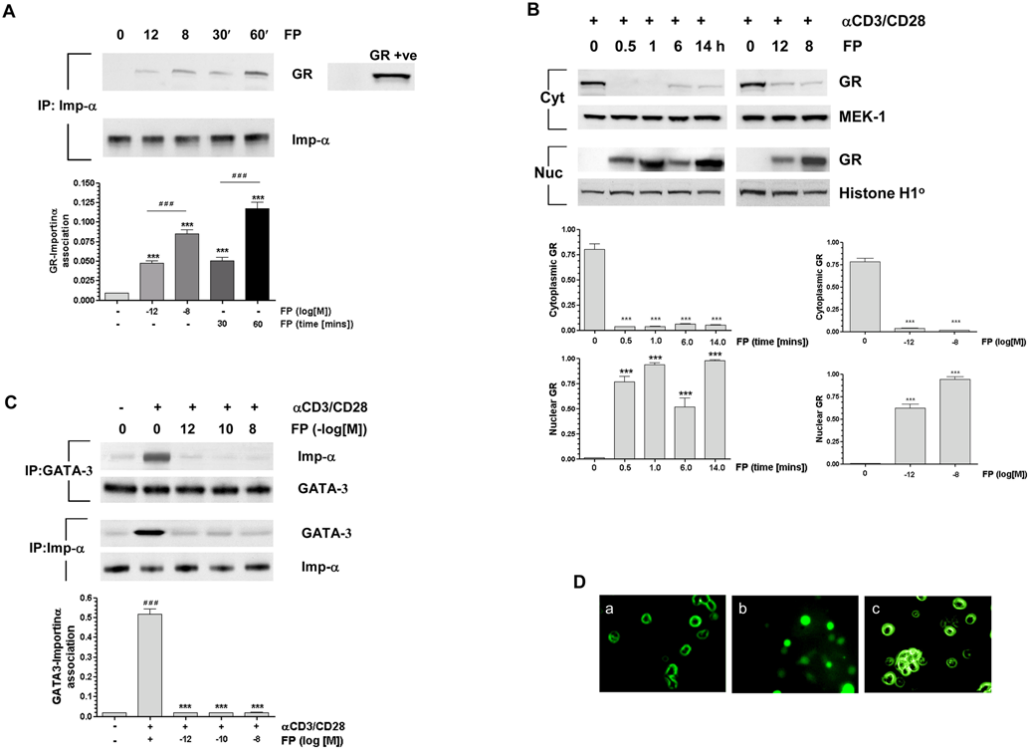
Fluticasone propionate reduces GATA-3 association with importin-α and GATA-3 nuclear import. (A) Western blot analysis demonstrates a time- (at 10^−8^ M FP) and concentration- (at 60 min after stimulation) dependent induction of FP-activated GR interaction with importin-α (Imp-α). A positive control for GR association with importin is shown. Quantification of the densitometry data is shown below. Each bar represents mean±SEM of at least three independent experiments. *** *p*<0.001 compared to control, ^###^ *p*<0.001. (B) Western blot analysis demonstrated a time- (at 10^−8^ M FP) and concentration- (at 60 min after stimulation) dependent induction of FP-activated GR nuclear translocation measured by IP. Quantification of the densitometry data is shown below. Each bar represents mean±SEM of at least three independent experiments. ***p<0.001 compared to control. (C) Western blot analysis of HuT-78 cells treated with FP and anti-CD3/CD28 co-stimulation demonstrated a concentration-dependent decrease in GATA-3–importin-α association at 20 min. Quantification of the densitometry data is shown below. Each bar represents mean±SEM of at least three independent experiments. ^###^ *p*<0.001 compared to control, ***p<0.001 compared to αCD3/CD28-stimulated cells. (D) GFP-tagged GATA-3 was overexpressed and cells stimulated (b, c) or not (a) for 30 min with anti-CD3/CD28. The effect of 30 min pretreatment of cells with FP (10^−8^ M, c) is also shown. All data were analysed by ANOVA followed by Newman-Keuls post-test.

We confirmed and extended previous data [20] to show that ligand-activated GR as well as GATA-3 uses importin-α for its nuclear import (Figure 2A and 2B). This interaction between GR and importin-α was significant at concentrations as low as 10^−12^ M and was maximal with 10^−8^ M FP. Subsequent GR nuclear translocation was rapid and sustained at significant levels for at least 14 h (Figure 2B). Using IP-Western blotting we showed that FP at 10^−12^–10^−8^ M decreased the association between GATA-3 and importin-α induced by anti-CD3/CD28 stimulation in a concentration-dependent manner (Figure 2C). In addition, using GFP-labelled GATA-3 and confocal microscopy we demonstrated that GATA-3 nuclear import following anti-CD3/CD28 stimulation for 30 min was attenuated by pretreatment with FP (10^−8^ M) (Figure 2D).

### Effect on MKP-1

**Figure 3:**
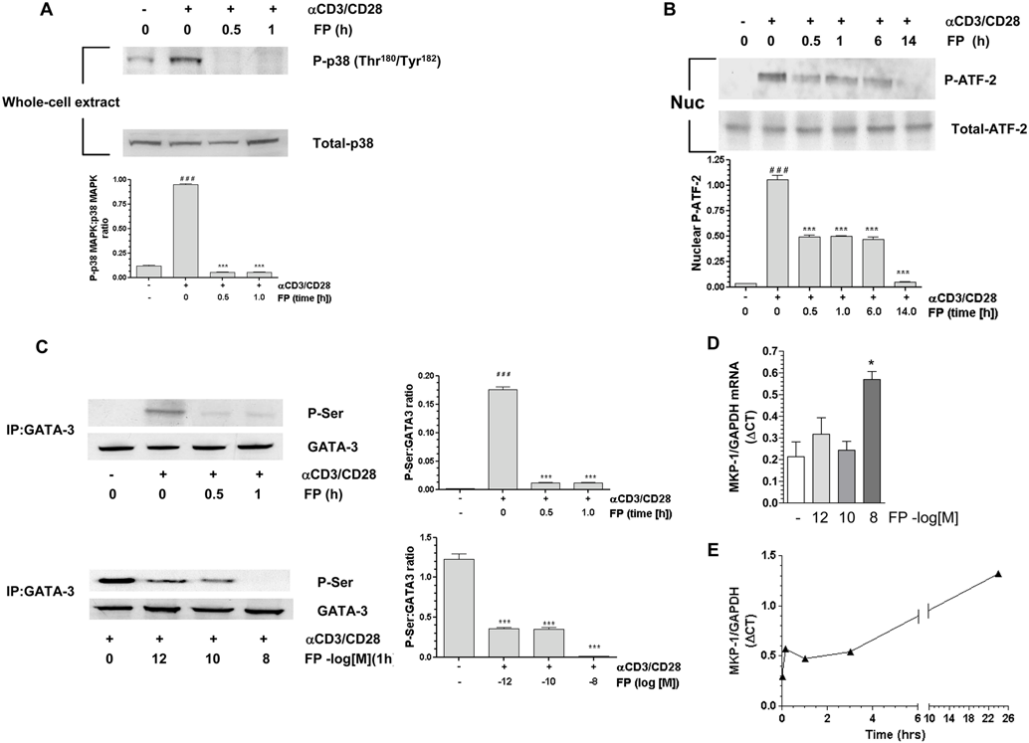
Fluticasone propionate–mediated inhibition of p38 MAP kinase phosphorylation and activation is associated with a marked down-regulation of GATA-3 serine phosphorylation. (A) Western blot analysis shows that FP (10^−8^ M, 30 min) treatment reduced dual phosphorylation (threonine-180 and tyrosine-182) of p38 MAPK in anti-CD3/CD28–co-stimulated HuT-78 cells. (B) Time course of the effect of FP (10^−8^ M) on phosphorylation of activated transcription factor 2 (ATF-2), a measure of p38 MAPK activity. (C) FP-induced inhibition of p38 MAPK activity is associated with the decrease of anti-CD3/CD28 co-stimulation–induced serine phosphorylation (P-Ser) of GATA-3. For (A–C), quantification of the densitometry data is also shown. Each bar represents mean±SEM of at least three independent experiments. ^###^ *p*<0.001 compared to control, ***p<0.001 compared to αCD3/CD28-stimulated cells. (D) FP induced MKP-1 mRNA in a concentration-dependent manner. All results are representative of at least three independent experiments and where appropriate expressed as means±SEM, *p<0.05. (E) FP induces MKP-1 mRNA in a time-dependent manner. Results are representative of two independent experiments. All data except (E) were analysed by ANOVA followed by Newman-Keuls post-test.

Dexamethasone inhibits p38 MAPK function in a cell type–specific manner through the rapid induction of the dual kinase phosphatase MKP-1 (MAPK phosphatase-1), and this effect lasts for up to 24 h [28]. FP (10^−8^ M) treatment of HuT-78 cells activated by anti-CD3/CD28 in vitro significantly decreased p38 MAPK phosphorylation (Figure 3A) and activity measured by phosphorylation of the downstream target ATF-2 (Figure 3B). This effect was detected at 30 min and lasted for at least 14 h (Figure 3B). FP (10^−8^ M) also significantly reduced GATA-3 serine phosphorylation induced by anti-CD3/CD28 stimulation in both a time- and concentration-dependent manner (Figure 3C). This reduction in GATA-3 phosphorylation was also seen with lower concentrations of FP. We found that FP significantly induced MKP-1 mRNA in both a time- and concentration-dependent manner, reaching a plateau at 10^−8^ M after 10 min (Figure 3D and 3E). However, the effects of FP on GATA-3 nuclear import, importin-α association and IL-4 mRNA expression are seen at 10,000-fold lower concentrations (10^−12^ M, see Figure 2).

**Figure 4:**
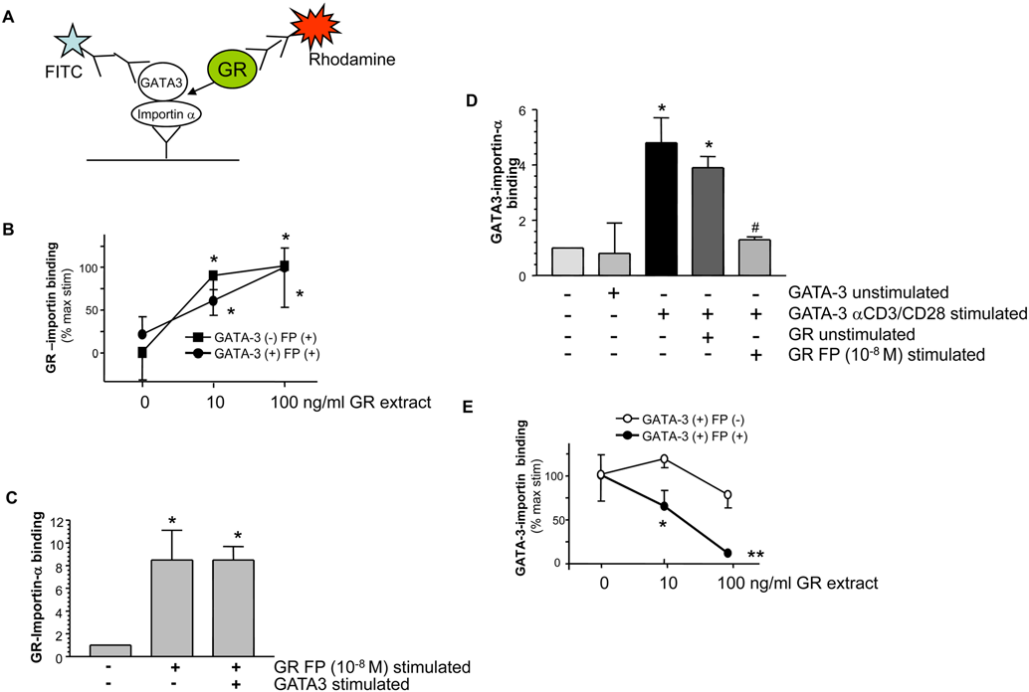
Fluticasone propionate competes with phospho-GATA-3 for importin-α. (A) schematic representation of the in vitro binding competition assay. (B) GR isolated from FP (10^−8^ M) stimulated cells enhances GR–importin-α binding in the presence (•) and absence (▪) of activated GATA-3. * *p*<0.05 compared to no activated GR. (C) GATA-3 isolated from anti-CD3/CD28–stimulated cells does not attenuate GR–importin-α association. *p<0.05 compared to control. (D) Activated GR blocks the ability of purified phospho-GATA-3 isolated from anti-CD3/CD28–stimulated cells interacting with immobilised importin-α in an in vitro binding assay. *p<0.05 compared to GATA-3 isolated from unstimulated cells. ^#^ *p*<0.05 compared to stimulated GATA-3-importin binding. (E) The effect of activated (•) versus unstimulated (○) GR on attenuation of GATA-3–importin-α association was concentration-dependent. *p<0.05, **p<0.01 between groups. All results are expressed as mean±SEM of three independent experiments and analysed by ANOVA followed by Newman-Keuls post-test.

Using an in vitro competition assay (Figure 4A) utilizing purified activated GATA-3, importin-α, and activated GR, we demonstrated that activated GR significantly increased GR-importin-α association in the presence and absence of activated GATA-3 (Figure 4B). This effect is not mutual, since activated GATA-3 did not block GR–importin-α association (Figure 4C). These data also suggest that both activated GR and phospho-GATA-3 can directly associate with importin-α (Figure 4D) and that activated GR attenuates the phospho-GATA-3/importin-α interaction in a concentration-dependent manner (Figure 4E). Together, this suggests that ligand-activated GR may compete with phospho-GATA-3 for importin-α and thereby limit GATA-3 nuclear import.

**Figure 5:**
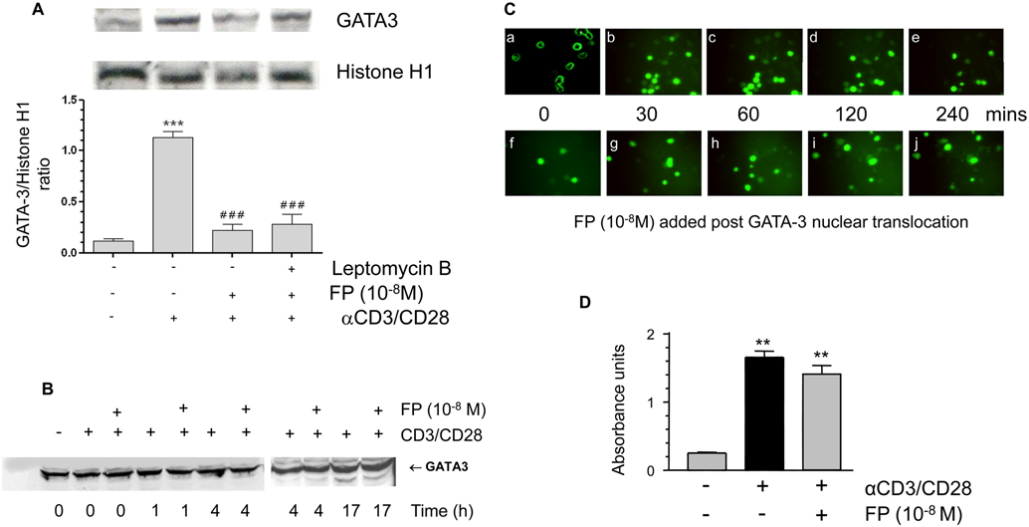
Fluticasone propionate does not affect GATA-3 nuclear export. (A) Western blot analysis showing that the nuclear export inhibitor leptomycin B (2 nM) does not affect the ability of FP (10^−8^ M) to prevent anti-CD3/CD28–stimulated GATA-3 nuclear localization measured at 60 min. ***p<0.001 compared to unstimulated cells, ^###^ *p*<0.001 compared to anti-CD3/CD28–stimulated cells. (B) Western blot analysis showing that FP (10^−8^ M) does not affect whole-cell GATA-3 degradation over 17 h. (C) GFP-tagged GATA-3 is overexpressed and cells stimulated (b–j) or not (a) with anti-CD3/CD28. The effect of treating cells with FP (10^−8^ M, f–j) after 30 min stimulation with anti-CD3/CD28 is also shown. (D) FP (10^−8^ M) does not prevent anti-CD3/CD28–stimulated p65 nuclear translocation at 60 min after stimulation. **p<0.01 compared to unstimulated cells. All results are representative of at least four independent experiments and are shown as mean±SEM. Results were analysed by ANOVA followed by Newman-Keuls test.

Other possible interpretations of our results could include an effect of FP on GATA-3 nuclear export and/or degradation. Leptomycin B, which inhibits nuclear export, did not affect the ability of FP to block GATA-3 nuclear localization (Figure 5A). Additionally, FP had no effect on whole cell GATA-3 expression during the time course of these experiments (Figure 5B). Nor did addition of FP subsequent to anti-CD3/CD28 nuclear translocation affect GATA-3 nuclear residency (Figure 5C), suggesting that activated GR does not enhance GATA-3 nuclear export. Finally, the effect of FP on GATA-3 nuclear import was not nonspecific, since FP (10^−8^ M) had no effect on p65 nuclear translocation measured at 60 min (Figure 5D).

### The Inhibitory Effect of Corticosteroids on GATA-3 Nuclear Localization in Primary T Lymphocytes Ex Vivo and In Vivo

**Figure 6:**
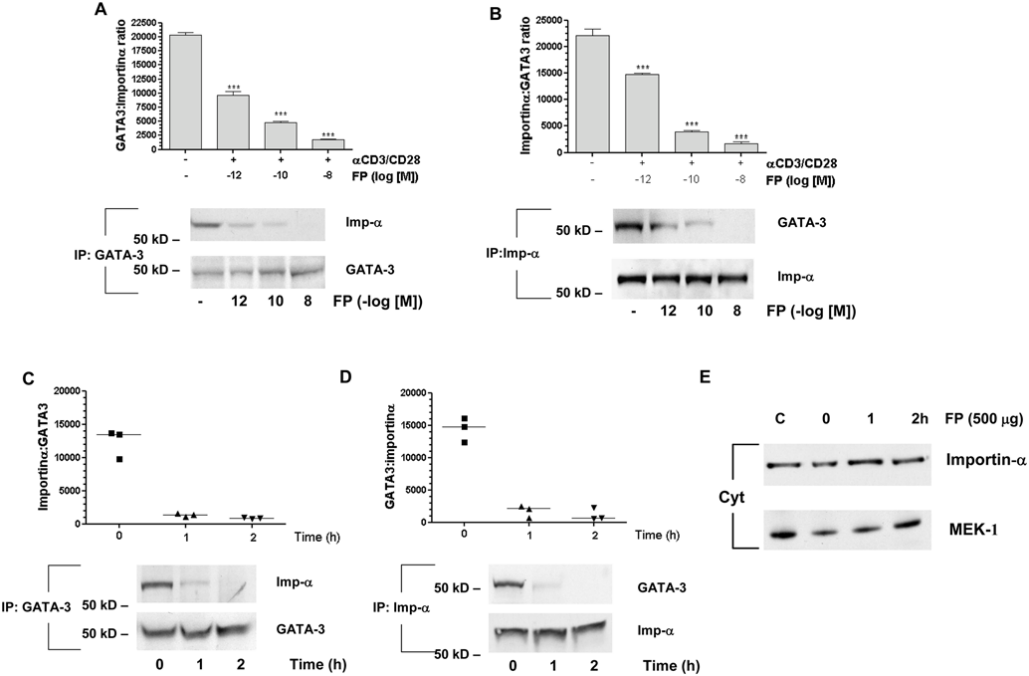
Fluticasone propionate impairs GATA-3 interaction with importin-α and GATA-3 nuclear localization in vivo and ex vivo. (A and B) Co-immunoprecipitation analysis of PBMCs from steroid-naïve asthma patients treated with FP in vitro demonstrated impaired interaction between GATA-3 and importin-α measured at 60 min. Each bar represents the mean±SEM of at least three independent experiments; *** *p*<0.001 compared with control as determined by ANOVA/Newman-Keuls analysis. (C and D) Co-immunoprecipitation analyses of PBMCs from steroid-naïve asthma patients treated with inhaled FP (500 µg via a spacer) in vivo demonstrated decreased association between GATA-3 and importin-α. The individual values for each treatment are presented graphically. (E) Representative Western blot showing that importin-α expression was unaffected by inhalation of FP. Blot is representative of gels from three participants.

Treatment with FP ex vivo demonstrated a concentration-dependent decrease in the direct interaction between phospho-GATA-3 and importin-α in PBMCs from patients with asthma (Figure 6A and 6B), which was significantly inhibited at 10^−12^ M FP (*p*<0.001, ANOVA and Newman-Keuls test) and completely attenuated by 10^−8^ M FP (*p*<0.001, ANOVA and Newman-Keuls test).

Our previous T cell line studies indicated that 10^−12^ M FP suppresses IL-4 and -5 gene expression and attenuated the interaction of GATA-3 with importin-α (see Figures 1D and 2). This concentration is close to peak plasma levels obtained from asthmatic patients treated with inhaled FP (500 µg) [27]. Inhaled FP (500 µg) treatment of seven steroid-naive asthma patients significantly reduced GATA-3–importin-α interaction in vivo in a time-dependent manner. This produced a >90% decrease in GATA-3–importin-α association at 2 h (median [95% CI], 13,494 [6,828–17,829] versus 879 [597–1,165]; *p*<0.05 Friedman's analysis). However, this did not reach significance using Wilcoxon's post-test analysis (W = 6.00) probably due to low numbers of participants. Similar results were observed when GATA-3–importin-α association was measured (Figure 6C and 6D). The lower dose of FP (100 µg) was not effective. The attenuated interaction of GATA-3 did not result from the defective recycling of importin-α, as a significant decrease in the abundance of importin-α in the cytoplasmic pool was not detected (Figure 6E).

**Figure 7:**
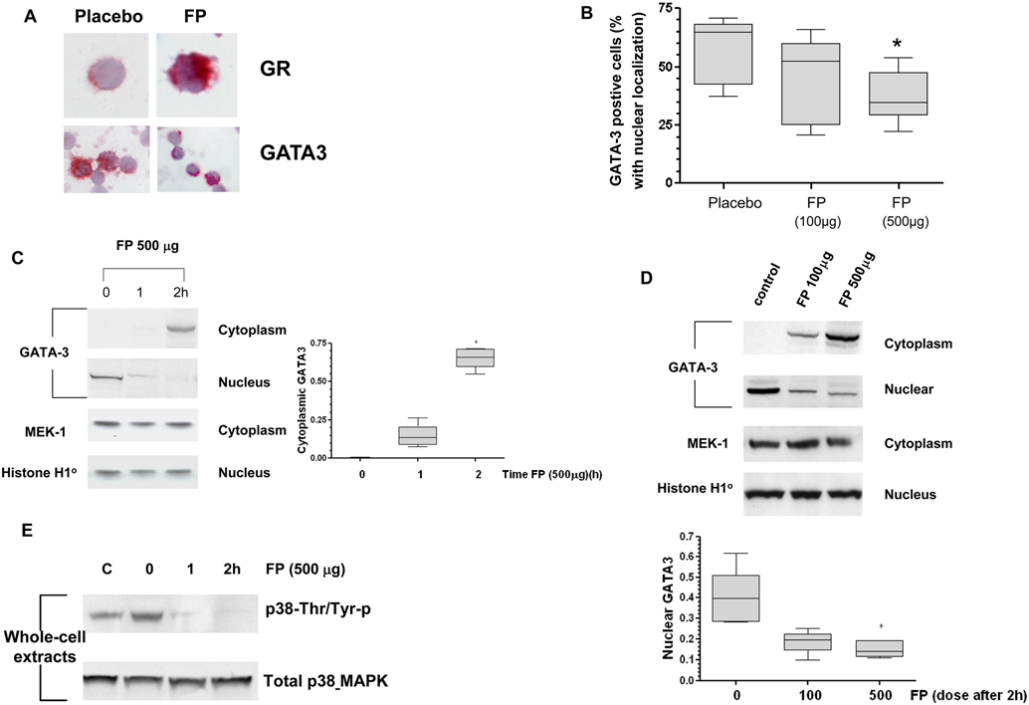
Inhaled fluticasone propionate impairs GATA-3 nuclear localization in PBMCs. (A) Representative immunocytochemistry of showing the effect of inhaled FP (500 µg) on GR and GATA-3 nuclear localisation. (B) Nuclear GATA-3 immunoreactivity in PBMCs from seven steroid-naïve asthma patients 2 h following inhaled FP treatment (100 or 500 µg via spacer). The median and interquartile ranges for each treatment are presented as a box-and-whiskers plot (*n* = 7); * *p*<0.05 Wilcoxon's rank test compared with placebo. (C) Immunoblotting analyses of PBMCs demonstrated a time-dependent decrease in nuclear expression of GATA-3, and increased cytoplasmic GATA-3 expression after inhalation of FP. (D) Immunoblotting analyses of PBMCs demonstrated a dose-dependent decrease in nuclear expression of GATA-3, and increased cytoplasmic GATA-3 expression 2 h after inhalation of FP. Histone H1 and MEK-1 immunoblotting confirmed equivalent total protein loading for the nuclear and cytoplasmic fractions respectively. Quantification of the densitometry data in (C) and (D) is shown as a box-and-whiskers plot of results from *n* = 6 participants for which data were available. *p<0.05 compared to control. (E) Western blot analyses of PBMCs demonstrated a time-dependent decrease in dual phosphorylation (threonine-180 and tyrosine-182) of p38 MAPK after inhalation of FP (500 µg). The results shown in (E) are representative of samples from two participants.

We further examined whether inhaled FP could affect cellular localization of GATA-3 in peripheral blood T cells. Treatment with inhaled FP (500 µg) for 2 h significantly increased GR nuclear translocation (Figure 7A) and concomitantly decreased the number of nuclear GATA-3 immunoreactive peripheral blood T cells (37%±4.2% versus 58.2%±4.95%, *p* = 0.016, W = 28.0, Wilcoxon's rank test) compared with placebo as measured by immunocytochemistry (Figure 7A and 7B). This was confirmed by Western blotting, which also indicated that this effect was both time- and dose-dependent (Figure 7C and 7D). Thus, inhaled FP (500 µg) induced significant loss in nuclear GATA-3 at 2 h (median [95% CI], 0.40 [0.27–0.53] versus 0.14 [0.11–0.19], *p*<0.05, W = 21.00, Wilcoxon's rank test) (Figure 7C) and cytoplasmic GATA-3 levels were enhanced by inhaled FP in a dose-dependent manner (median [95% CI], 0.0032 [0.0026–0.0039] versus 0.658 [0.592–0.720], *p*<0.05, W = −21.00, Wilcoxon's rank test) (Figure 7D). In addition, FP (500 µg) inhibited p38 MAPK phosphorylation in primary T cells in vivo at 2 h in samples from two patients (Figure 7E).

Taken together, our data suggest that inhaled FP reduces nuclear localization of GATA-3 in vivo by acutely inhibiting phospho-GATA-3–importin association. This effect may be direct, through competition for importin-α or associated molecules, or secondary to an effect on p38 MAPK-mediated GATA-3 phosphorylation via rapid induction of MKP-1. The combination of these two interacting effects can result in complete suppression of GATA-3 nuclear import and thus Th2 cytokine gene expression.

## Discussion

Here we have demonstrated, to our knowledge for the first time, that corticosteroids may inhibit GATA-3 function and therefore the transcription of Th2 genes via two distinct but interacting molecular mechanisms. Firstly, corticosteroid-activated GR appears to compete with activated GATA-3 for nuclear import via importin-α, which is required for the nuclear transport of both GATA-3 and GR. Secondly, corticosteroids at higher concentrations increase the expression of MKP-1, a potent inhibitor of p38 MAPK activity and thereby prevent T cell receptor/co-receptor activation of p38 MAPK to prevent the phosphorylation of GATA-3 that is necessary for interaction with importin-α and subsequent nuclear import.

We have previously shown that translocation of GATA-3 from the cytoplasm to the nucleus involves the nuclear transporter protein importin-α, which interacts with phosphorylated GATA-3 [12]. We have also previously reported that GATA-3 knockdown using siRNA results in suppression of anti-CD3/CD28–stimulated IL-4/IL-5 mRNA induction, thus implicating an essential role for GATA-3 in the transcription of these genes [12]. We now confirm, in human T cells, that GR also uses the same nuclear import mechanism as GATA-3 [22]. We therefore propose that there is competition between ligand-activated GR and phospho-GATA-3 for nuclear import. Furthermore, we have shown that there is preferential binding of importin-α to activated GR over phospho-GATA-3, so that corticosteroids would preferentially reduce GATA-3 entry and thus rapidly switch off Th2 gene transcription without any need for any intermediate steps. Furthermore, there was some degree of specificity for GATA-3, as nuclear translocation of the p65 subunit of NF-κB was not affected by corticosteroid exposure.

We tested some alternative explanations for this effect of FP on GATA-3 nuclear exclusion and failed to show that FP either enhances GATA-3 nuclear export directly or induces GATA-3 degradation. The evidence from the in vitro competition assays does, however, suggest that purified activated GR can clearly attenuate purified phospho-GATA-3–importin-α association and that the converse does not occur. Furthermore, we have shown that only phospho-GATA-3 can associate with importin-α. This mechanism is sensitive to very low concentrations of corticosteroid and would be rapid in onset as no changes in protein synthesis are required. This acute mechanism may also contribute to the reduction in GATA-3 nuclear import and may play a major role at low corticosteroid concentrations and/or at early time points prior to MKP-1 induction.

Corticosteroids can modulate p38 MAPK activity through the induction of MKP-1, a potent endogenous inhibitor of MAPK function [38],[39]. We report here a rapid induction of MKP-1 mRNA following stimulation of cells with relatively high concentrations of FP. We hypothesize that this rapid induction of MKP-1 can reduce GATA-3 nuclear import by attenuating p38 MAPK activity and subsequent GATA-3 phosphorylation, thus preventing nuclear translocation. The location of the serine residue(s) of GATA-3 that are phosphorylated by p38 MAPK are currently unknown, but a bioinformatics search (Motif Scanner, http://scansite.mit.edu/motifscan_seq.phtml) indicates at least three potential p38 MAPK-sensitive serine residues.

As predicted from these in vitro data, impairment of GATA-3 nuclear import by FP may, at least in part, underlie the efficacy of corticosteroids in suppressing allergic inflammation. Although we did not assess the acute inhibitory effect of FP on the expression of IL-4 mRNA in vivo, a single inhalation of FP (500 µg) may have comparable effects, as it provides plasma levels within a relevant range of concentrations used to suppress IL-4 transcription in our in vitro system [37]. A lower dose of inhaled FP (100 µg) was not effective, but plasma concentrations may be below those required for GATA-3 inhibition. However, it is likely that the higher concentrations of FP in the airways after inhaled administration would be effective in inhibiting GATA-3 in airway T cells of asthma patients. The study of PBMCs from asthma patients treated with inhaled corticosteroid therapy clearly demonstrates that these molecular mechanisms are likely to also occur in patients at therapeutic doses of inhaled corticosteroids. In addition, previous studies have shown that corticosteroids can suppress IL-4 and IL-5 release from peripheral blood cells of asthma patients in vitro and in vivo [40]–[42].

In summary, our data provide evidence for a novel action of corticosteroids: suppression of allergic inflammation through a rapid inhibitory effect on GATA-3 nuclear translocation by preferential binding to the shared nuclear import protein importin-α and by a second mechanism involving increased synthesis of MKP-1, which inhibits p38 MAPK, thus preventing the phosphorylation of GATA-3 that is necessary for nuclear translocation of GATA-3. These two mechanisms are likely to be synergistic, accounting for the rapid and potent effect of corticosteroids on allergic inflammation. This is exemplified by the rapid inhibitory effect of topical corticosteroids on nasal Th2 cytokine release after allergen provocation in individuals with seasonal allergic rhinitis (hay fever) [43]. Prevention of phospho-GATA-3 interaction with importin-α may provide a new approach for the development of novel therapies for the treatment of allergic diseases.

## Abbreviations

FP: fluticasone propionate
GR: glucocorticoid receptor
GRE: glucocorticoid response element
IP: immunoprecipitation
NLS: nuclear localisation sequence(s)
PBMC: peripheral blood mononuclear cell
RT-PCE: reverse transcription PCR
SEM: standard error of the mean
Th2: T helper-2
