# Correction: Suppression of GATA-3 Nuclear Import and Phosphorylation: A Novel Mechanism of Corticosteroid Action in Allergic Disease

**DOI:** 10.1371/journal.pmed.1002657

**Published:** 2018-09-07

**Authors:** Kittipong Maneechotesuwan, Xin Yao, Kazuhiro Ito, Elen Jazrawi, Omar S. Usmani, Ian M. Adcock, Peter J. Barnes

The *PLOS Medicine* Editors received comments from readers suggesting that the Western blot demonstrating nuclear translocation of GATA-3 in response to anti-CD3/CD28 stimulation in panel A of Fig 1 was labeled incorrectly. The authors have re-examined the original experimental images and would like to correct Fig 1. Please see the corrected version of panel A in Fig 1 below. The conclusions of the paper are unaffected by this correction.

**Fig 1:**
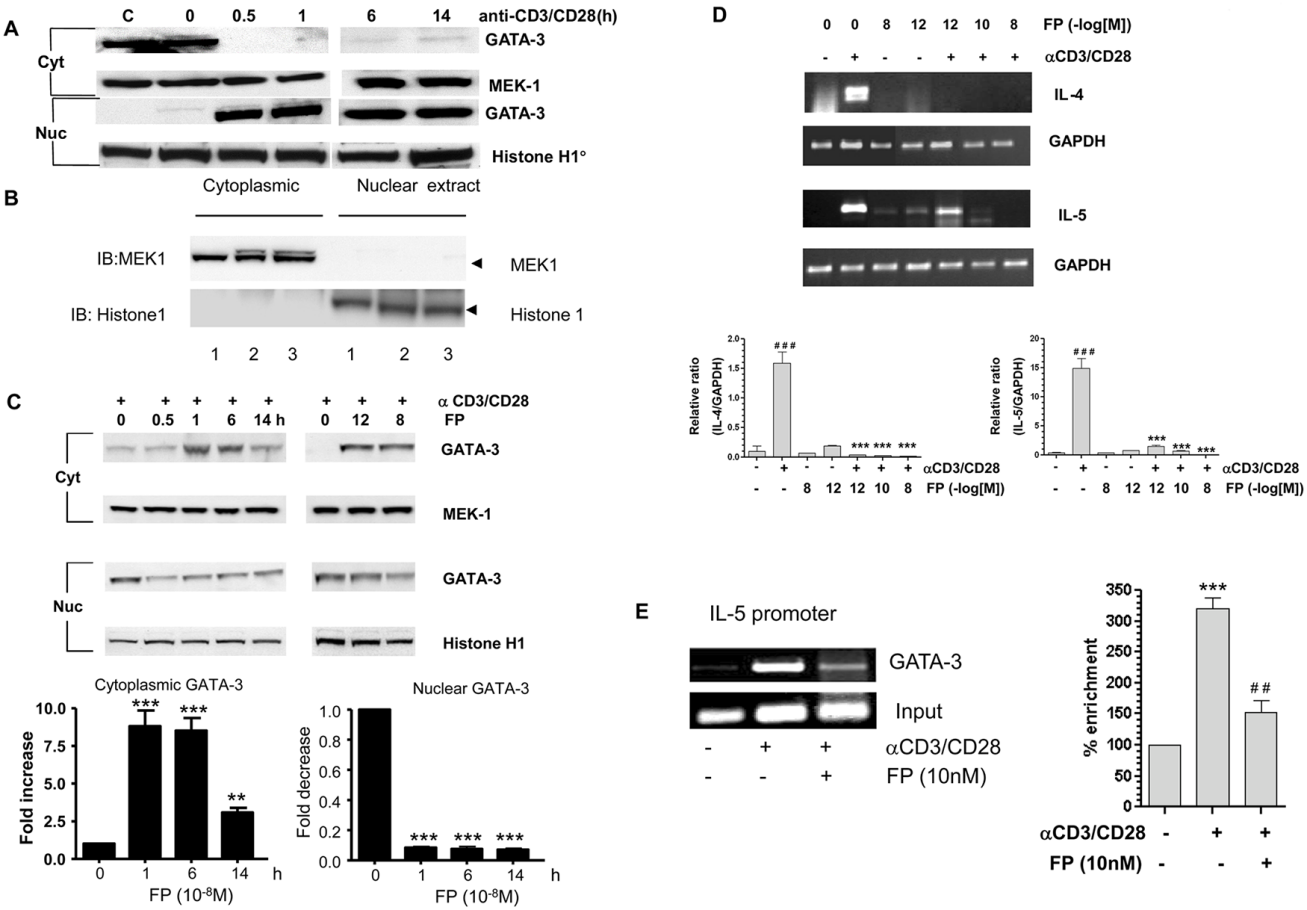
Fluticasone propionate down-regulates Th2 cytokine gene expression and inhibits GATA-3 nuclear import. (A) Anti-CD3/CD28 treatment of HuT-78 cells results in translocation of GATA-3 from the cytoplasm to the nucleus within 30 min. (B) Histone H1 and MEK-1 were used to confirm distinct separation of cytoplasmic and nuclear extracts in three separate experiments. (C) Western blot analysis of FP-treated HuT-78 cells demonstrated impaired nuclear localization of GATA-3 induced by anti-CD3/CD28 co-stimulation in a time- (at 1028 M FP) and concentration- (at 60 min after stimulation) dependent manner. Cells were pretreated with FP for 30 min prior to stimulation. MEK1 and histone H1 were used to demonstrate equal cytoplasmic and nuclear loading respectively. Results are presented graphically below as mean6SEM of at least three independent experiments. *** p,0.001 compared to t = 0. (D) RT-PCR showing that FP inhibits IL-4 and IL-5 mRNA expression in CD3/CD28- costimulated cells. GAPDH was used as a loading control. Lower panels show graphical analysis of results presented as mean6SEM of at least three independent experiments. ###p,0.001 compared to control, ***p,0.001 compared to anti-CD3/CD28–stimulated. (E) FP (10 nM) reduces the ability of anti-CD3/CD28-stimulated GATA-3 to associate with the native IL-5 promoter 60 min after stimulation. Data are also shown graphically as mean6SEM of three independent experiments. All data were analysed by ANOVA followed by Newman-Keuls post-test.
